# Expression of Notch1 and Numb in small cell lung cancer

**DOI:** 10.18632/oncotarget.14411

**Published:** 2017-01-02

**Authors:** Hajime Kikuchi, Jun Sakakibara-Konishi, Megumi Furuta, Hiroshi Yokouchi, Hiroshi Nishihara, Shigeo Yamazaki, Hidetaka Uramoto, Fumihiro Tanaka, Masao Harada, Kenji Akie, Fumiko Sugaya, Yuka Fujita, Kei Takamura, Tetsuya Kojima, Toshiyuki Harada, Mitsunori Higuchi, Osamu Honjo, Yoshinori Minami, Naomi Watanabe, Satoshi Oizumi, Hiroyuki Suzuki, Takashi Ishida, Hirotoshi Dosaka-Akita, Hiroshi Isobe, Mitsuru Munakata, Masaharu Nishimura

**Affiliations:** ^1^ Department of Medicine, Hokkaido University School of Medicine, Sapporo, Japan; ^2^ Department of Pulmonary Medicine, Fukushima Medical University, Fukushima, Japan; ^3^ Department of Translational Pathology, Hokkaido University Graduate School of Medicine, Sapporo, Japan; ^4^ Department of Thoracic Surgery, Keiyukai Sapporo Hospital, Sapporo, Japan; ^5^ Department of Surgery, University of Occupational and Environmental Health, Kita-kyushu, Japan; ^6^ Department of Thoracic Surgery, Kanazawa Medical University, Uchinada, Japan; ^7^ Department of Respiratory Medicine, National Hospital Organization Hokkaido Cancer Center, Sapporo, Japan; ^8^ Department of Respiratory Disease, Sapporo City General Hospital, Sapporo, Japan; ^9^ Department of Respiratory Medicine, Teine Keijinkai Hospital, Sapporo, Japan; ^10^ Department of Respiratory Medicine, National Hospital Organization Asahikawa Medical Center, Asahikawa, Japan; ^11^ Department of Medicine, Obihiro Kosei Hospital, Obihiro, Japan; ^12^ Department of Medical Oncology, KKR Sapporo Medical Center, Sapporo, Japan; ^13^ Center for Respiratory Diseases, JCHO Hokkaido Hospital, Sapporo, Japan; ^14^ Department of Thoracic Surgery, Fukushima Red Cross Hospital, Fukushima, Japan; ^15^ Department of Thoracic Surgery, Fukushima Medical University, Fukushima, Japan; ^16^ Department of Respiratory Medicine, Sapporo-Kosei General Hospital, Sapporo, Japan; ^17^ Respiratory Center, Asahikawa Medical University, Asahikawa, Japan; ^18^ Department of Internal Medicine, Sunagawa City Medical Center, Sunagawa, Japan; ^19^ Clinical Oncology Center, Fukushima Medical University Hospital, Fukushima, Japan; ^20^ Department of Medical Oncology, Hokkaido University Graduate School of Medicine, Sapporo, Japan

**Keywords:** small cell lung cancer, Notch1, Numb, immunohistochemistry, surgery

## Abstract

Notch signaling in tumorigenesis functions as an oncogene or tumor suppressor according to the type of malignancy. Numb represses intracellular Notch signaling. Previous studies have demonstrated that Notch signaling suppresses the proliferation of small cell lung cancer (SCLC) cell lines. However, in SCLC, the association between Notch1 and Numb expression and clinicopathological factors or prognosis has remained unclear. In this study, we evaluated the expression of Notch1 and Numb in SCLC. We immunohistochemically assessed 125 SCLCs that were surgically resected at 16 institutions participating in either the Hokkaido Lung Cancer Clinical Study Group Trial (HOT) or the Fukushima Investigative Group for Healing Thoracic Malignancy (FIGHT) between 2003 and 2013. Correlations between Notch1 or Numb expression and various clinicopathological features were evaluated. Notch1 expression was associated with ECOG performance status. Numb expression was associated with age, sex, and pathological histology (SCLC or Combined SCLC). Analysis of cellular biological expression did not demonstrate a significant correlation between the expression of Notch1 and of Numb. Multivariate Cox regression analysis showed that high Notch1 expression was an independent favorable prognostic factor for SCLC(hazard ratio = 0.503, P = 0.023). High Notch1 expression, but not Numb expression, is associated with favorable prognosis in SCLC.

## INTRODUCTION

Lung cancer is the leading cause of cancer-related death worldwide, and small cell lung cancer (SCLC) accounts for approximately 13–15% of new lung cancers each year [1]. SCLC is characterized by an extremely aggressive behavior with early systemic spreading and the prognosis of patients with SCLC remains poor and further treatment is required [2].

The Notch signaling pathway regulates many fundamental processes essential for normal development such as the control of cell differentiation, survival, proliferation, and angiogenesis. In mammals, there are four Notch receptors (Notch1 to Notch4) and two families of ligands, Jagged (JAG1, 2) and Delta-like ligands (DLL1, 3, 4). Binding of Notch ligands induces proteolytic cleavage of Notch receptor by γ-secretase, thereby releasing the Notch intracellular domain (NICD) into the cytoplasm. The NICD then enters the nucleus, interacts with the transcription factor CBF1/Suppressor of Hairless/Lag1 (CSL), and induces the transcription of several target genes, including *hairy enhancer of split 1* (*HES1*) [3, 4].

Notch signaling in tumorigenesis functions as an oncogene or tumor suppressor according to the type of malignancy [3, 4]. We previously showed that inhibition of Notch signaling by a γ-secretase inhibitor suppresses the growth of non-small cell lung cancer (NSCLC) [5]. Gain-of-function mutations of the *NOTCH1* gene are present in about 10% of NSCLC, and activation of Notch1 correlates with poor clinical outcomes in NSCLC patients [6]. Regarding SCLC, it has been reported that overexpression of Notch1 induces G1 cell cycle arrest and inhibits cell growth, epithelial mesenchymal transition (EMT), cell invasion, and metastasis, *in vitro* and *in vivo* [7, 8, 9]. However, the relationship between Notch1 expression and clinicopathological factors in SCLC has not been reported.

Numb is a negative regulator of Notch and determines cell fate [10]. Numb interacts with the E3 ubiquitin ligase, Itch, to ubiquitinate Notch receptors and NICD, and Numb interferes with the nuclear translocation of NICD through direct binding to it [11, 12]. Although experimental evidence indicates that Numb has a potential function as a tumor suppressor in many cancers, the role of Numb in SCLC has not been determined.

We hypothesized that Notch1 or Numb expression might be correlated with prognosis in SCLC patients. In the present study, we analyzed the expression of Notch1 and Numb in surgically-resected SCLCs by immunohistochemistry and evaluated correlations between their expression and various clinicopathological features.

## RESULTS

### Notch1 and Numb expression in SCLC

**Figure 1:**
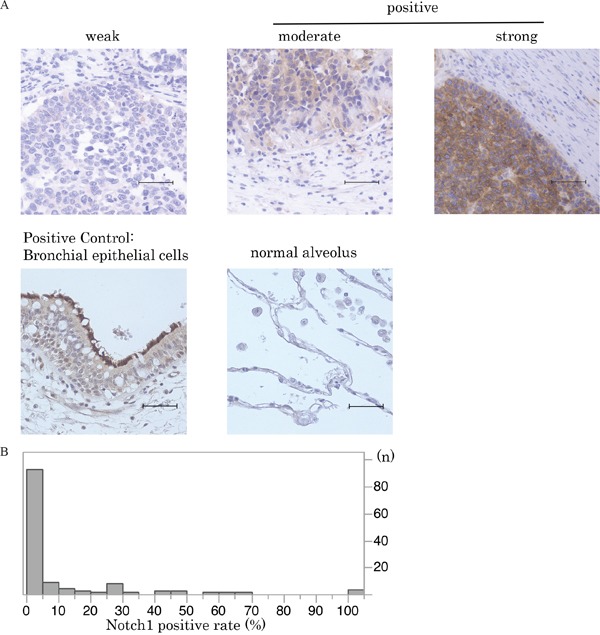
Notch1 expression in SCLC **A**. Immunohistochemical staining patterns of Notch1 are shown in SCLC. SCLC specimens were stained with anti-Notch1 antibody. Notch1 expression in normal bronchial and alveolar cells is also shown (scale bar = 50 μm). **B**. This histogram illustrates the distribution of Notch1 expression of any intensity in the 125 SCLC specimens that were investigated in the current study. The median Notch1-positive staining rate in SCLC tumors was 0% (95%CI:0-0.6). Cases with more than 5% staining were defined as the high Notch1 expression group.

Normal bronchial epithelial cells were used as positive controls of both Notch1 and Numb expression in immunohistochemical analyses. Weak expression of both Notch1 and Numb was observed in normal alveoli (Figure 1A, 2A). Once processed by γ-secretase, the Notch1 ICD is released from the plasma membrane and translocates into the nucleus, where it activates target gene transcription. Therefore, only membrane staining was considered as negative and cytoplasm and/or nuclear staining were considered as positive staining in the evaluation of activated Notch1 expression (Figure 1A) [6, 13]. In present study, there was no case with only membrane staining.

**Figure 2:**
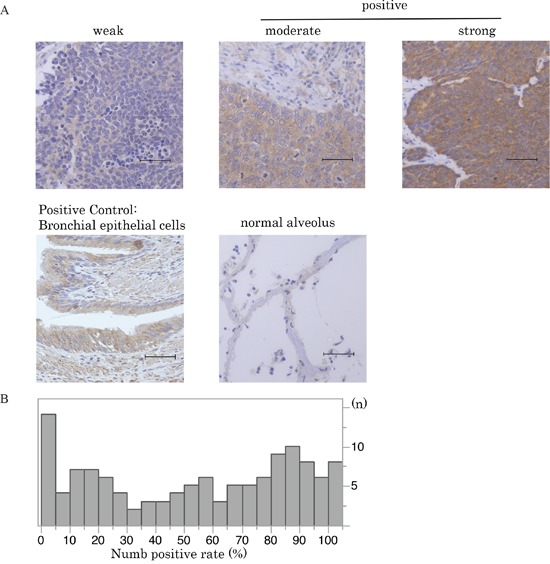
Numb expression in SCLC **A**. Immunohistochemical staining patterns of Numb are shown in SCLC. SCLC specimens were stained with anti-Numb antibody. Numb expression in normal bronchial and alveolar cells is also shown (scale bar = 50 μm). **B**. This histogram illustrates the distribution of Numb expression of any intensity in the 125 SCLC specimens that were investigated in the current study. The median Numb-positive staining rate in SCLC tumors was 57.8% (95%CI:46.6-70.8). Cases with higher than the median positive rate were defined as the high Numb expression group.

The distribution of the degree of Notch1 expression in the study cohort is shown in Figure 1B (range, 0–100%). The median Notch1-positive staining rate in SCLC tumors was 0% (95%CI:0-0.6). We thus defined cases with more than 5% staining as the Notch1 high expression group. High Notch1 expression was observed in 33 cases (26.4%).

Numb immunoreactivity was present predominantly in the cytoplasm of tumor cells (Figure 2A). The distribution of the degree of Numb expression in the study cohort is shown in Figure 2B (range, 0–100%). The median Numb-positive staining rate in SCLC tumors was 57.8% (95%CI:46.6-70.8). We defined cases with higher than the median positive rate as the Numb high expression group, which included 63 patients (50.4%).

### Correlation between Notch1 or Numb expression and clinical and clinicopathological characteristics

High expression of Notch1 was significantly more prevalent in patients with Eastern Cooperative Oncology Group performance status (ECOG PS) 0 than 1 (P< 0.05; Fisher exact tests), whereas no other clinical or clinicopathological parameters were correlated with Notch1 expression (Table 1).

**Table 1:** Relationship between the expression of Notch1 or Numb and clinical and clinicopathological characteristics in 125 cases with surgically resected small cell lung cancer

Characteristics	Notch1 expression			Numb expression		
	No. of Patients			No. of Patients		
	Low	High	*P*	Low	High	*P*
**Age (years)**						
<65	28	10	1.000	25	13	0.032
≧65	64	23		38	49	
**Sex**						
Male	70	25	1.000	41	54	0.006
Female	22	8		22	8	
**Smoking**						
Smoker	80	28	0.457	53	55	0.323
Nonsmoker	6	4		7	3	
Unknown	6	1		3	4	
**Smoking (pack-years)**						
≧20	76	28	1.000	50	54	0.154
<20	10	4		10	4	
Unknown	6	1		3	4	
**ECOG PS^a^**						
0	59	29	0.004	46	42	0.836
1	28	2		15	15	
Unknown	5	2		2	5	
**Histlogy**						
SCLC^b^	6721	0.376	51	37	0.011	
Combined SCLC	25	12		12	25	
**pT^c^**						
T1	54	16	0.414	32	38	0.281
T2-4	38	17		31	24	
**pN^d^**						
N0	60	21	0.833	42	39	0.706
N1-3	31	12		20	23	
**pStage^e^**						
I	54	20	1.000	37	37	1.000
II-IV	38	13		26	25	
**Adjuvant chemotherapy**						
Yes	55	21	0.837	38	38	1.000
No	35	12		23	24	
Unknown	2	0		2	0	Eastern

^a^ Eastern Cooperative Oncology Group performance status.

^b^ Small cell lung cancer.

^c^ Pathological tumor classification.

^d^ Pathological lymph node status.

^e^ Pathological disease stage.

Fisher exact tests indicated that high Numb expression was significantly more prevalent in tumors from patients ≧65 years old versus tumors from patients <65 years old (P < 0.05); in tumors from men versus tumors from women (P < 0.05); and in combined SCLC versus SCLC (P < 0.05). Numb expression was not associated with smoking history, ECOG PS, pathological tumor (pT) status, pathological lymph node (pN) status or pathological Stage (Table 1).

Analysis of cellular biological expression showed that there was no statistically significant correlation between the expression of Notch1 and Numb (Table 2). Moreover, we obtained the data about the expression of neuroendocrine (NE) -specific proteins, including ChromograninA, Synaptophysin, and CD56, based on the pathological reports of each surgical specimen as possible. There were no significant correlations between the expression of Notch1 or Numb and the expression of NE-specific proteins (Table 3).

**Table 2:** Relationship between the expression of Notch1 and Numb

	Notch1 expression		
	Low	High	*P*
Numb expression			
Low	51	12	0.070
High	41	21	

**Table 3:** Relationship between the expression of Notch1 or Numb and Neuroendocrine-specific protein expression

NE^a^-specific protein	Notch1 expression			Numb expression		
	No. of Patients			No. of Patients		
	Low	High	*P*	Low	High	*P*
**ChromograninA**						
Positive	27	8	1.000	15	20	1.000
Negative	21	6		12	15	
Unknown	44	19		36	27	
**Synaptophysin**						
Positive	33	10	0.479	24	19	0.394
Negative	15	2		7	10	
Unknown	44	21		31	34	
**CD56**						
Positive	43	13	0.568	24	32	1.000
Negative	4	0		2	2	
Unknown	45	20		37	28	

^a^ Neuroendocrine.

### Prognostic value of Notch1 or Numb expression in SCLC

**Figure 3:**
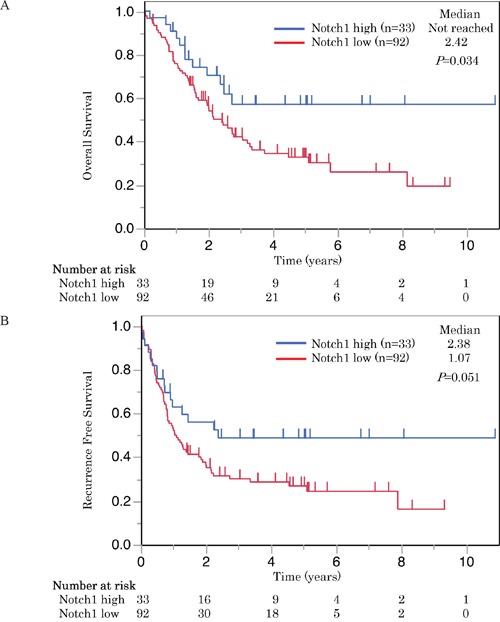
These Kaplan-Meier survival curves illustrate **A**. overall survival and **B**. recurrence free survival of patients with SCLC who underwent radical resection. Survival curves were stratified by the expression of Notch1 for all patients with SCLC (n = 125). High Notch1 expression was correlated with longer overall survival and also tended to have longer recurrence free survival in SCLC patients.

**Figure 4:**
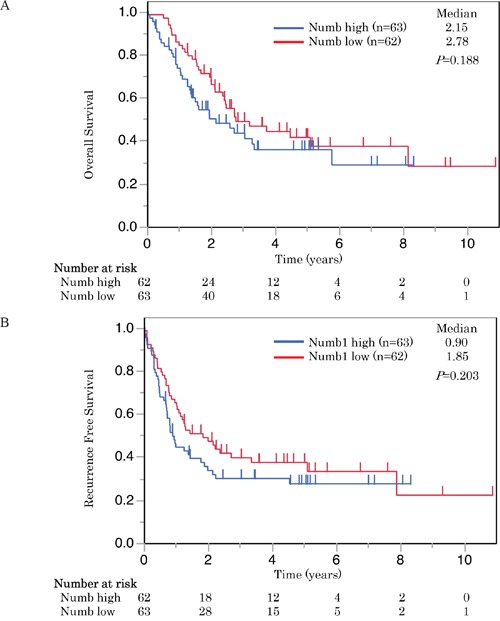
These Kaplan-Meier survival curves illustrate **A**. overall survival and **B**. recurrence free survival of patients with SCLC who underwent radical resection. Survival curves were stratified by the expression of Numb for all patients with SCLC (n = 125). Numb expression had no impact on survival.

Patients with high Notch1 expression had significantly longer overall survival (OS) than those with low expression (P < 0.05) (Figure 3A). The cases with high expression of Notch1 also had a longer recurrence-free survival (RFS) than those with low expression, but the difference was not statistically significant (P = 0.051) (Figure 3B). On the other hand, Numb expression was not associated with either OS or RFS (Figure 4A, 4B).

Next, the importance of Notch1 as a prognostic factor of OS was analyzed in a Cox proportional hazards model analysis. In univariate analysis of potential prognostic factors, low expression of Notch1, advanced pT classifications, and no adjuvant chemotherapy were significant, unfavorable prognostic factors (Table 4). In multivariate analysis, low expression of Notch1, advanced pT classification, and no adjuvant chemotherapy were significant and independent, unfavorable prognostic factors (P < 0.05) (Table 4).

**Table 4:** Univariate and multivariate analyses using the Cox proportional hazards model for clinical and clinicopathological factors that affected overall survival after small cell lung cancer (SCLC) resection for SCLC

	Univariate analysis		Multivariate analysis	
	HR^a^ (95%C.I.^b^)	*P*	HR (95%C.I.)	*P*
**Age (years)**				
≧65 vs. <65	1.035 (0.634-1.746)	0.893		
**Sex**				
Male vs. Female	1.435 (0.833-2.633)	0.200		
**Smoking history (pack-years)**				
≧20 vs. <20	1.107 (0.561-2.513)	0.785		
**ECOG PS^c^**				
1 vs. 0	1.618 (0.934-2.703)	0.084		
**Histlogy**				
SCLC^d^ vs.	0.948 (0.574-1.627)	0.840		
Combined SCLC				
**pT^e^**				
T2-4 vs. T1	1.666 (1.041-2.671)	0.034	1.812 (1.115-2.947)	0.017
**pN^f^**				
N1-3 vs. N0	1.508 (0.920-2.428)	0.102		
**Adjuvant chemotherapy**				
Yes vs. No	0.524 (0.325-0.854)	0.010	0.546 (0.338-0.892)	0.016
**Notch1**				
High vs. Low	0.517 (0.265-0.927)	0.026	0.503 (0.255-0.913)	0.023
**Numb**				
High vs. Low	1.367 (0.856-2.186)	0.190		

^a^ Hazard ratio.

^b^ Confidence interval.

^c^ Eastern Cooperative Oncology Group performance status.

^d^ Small cell lung cancer.

^e^ Pathological tumor classification.

^f^ Pathological lymph node status.

## DISCUSSION

In the current study, we demonstrated that Notch1 expression was associated with ECOG PS and that Numb expression was associated with age, sex, and pathological histology. There was a positive correlation between high Notch1 expression and favorable patient prognosis in SCLC. To our knowledge, this is the first study to examine the association between Notch1 or Numb expression and clinicopathological factors or prognosis in patients with SCLC.

High Notch1 expression is an independent poor prognostic factor in many other cancers, such as breast and gastric cancer [14, 15, 16]. NSCLC patients with high Notch1 expression showed poor survival as well [6, 17]. However, there is another report which demonstrated a divergent impact of Notch1 expression based on histological subtypes, adenocarcinoma and squamous cell carcinoma [13]. It is well known that the pleiotropic functions of Notch can be tumor suppressive or oncogenic depending on the cellular context [4]. In our study, high Notch1 expression was a favorable prognostic factor in patients with SCLC. It has been reported that the overexpression of Notch1 in SCLC cell lines induces G1 cell cycle arrest and inhibits cell growth, EMT, cell invasion, and metastasis, *in vitro* and *in vivo* [7, 8, 9]. Recently, comprehensive genomic profiles of human SCLC were analyzed and the most damaging mutations were observed in the extracellular domain of Notch receptors, suggesting that *NOTCH* genes are tumor suppressors in SCLC [18]. Our findings are consistent with previous reports regarding the tumor suppressive role of Notch1 in SCLC.

Loss of Numb expression has been reported in some types of human cancers, such as breast cancers and NSCLC [6, 19]. In esophageal carcinoma and malignant pleural mesothelioma, overexpression of Numb suppresses tumor cell growth and loss of Numb expression is associated with poor prognosis [20, 21]. Although most previous reports regarding the role of Numb in tumorigenesis suggest that Numb functions as a tumor suppressor, there have been few reports that have investigated the function of Numb in lung cancer. In the present study, Numb expression was not associated with prognosis in SCLC patients. Numb contains an amino-terminal phosphotyrosine-binding (PTB) domain and C-terminal proline-rich (PRR) and Eps15 homology regions. It has been reported that an increased level of the Numb PRR long (PRR^L^) isoform promotes cell proliferation, whereas the PRR short (PRR^S^) isoform induces cell differentiation [22, 23]. Although we evaluated pan Numb expression in the present study, the expression of each Numb isoform should probably be investigated in SCLC.

Although an inverse correlation between Notch1 expression and Numb expression was previously observed in NSCLC [6], in the present study there was no statistically significant correlation between the expression of Notch1 and Numb. It has been reported that increased expression of the Numb PRR^S^ isoform suppresses Notch signaling and decreases Notch target gene expression such as *HES1* in lung cancer cells, whereas increased expression of the PRR^L^ isoform antagonizes PRR^S^ activity leading to increased Notch target gene expression [24]. The opposite role of Numb isoforms in regulation of the Notch pathway may mask relationships between Notch and Numb.

In conclusion, high Notch1 expression, but not Numb expression, is correlated with favorable prognosis in SCLC patients. Notch1 provides a novel prognostic marker of SCLC and activation of Notch1 may contribute to the development of a new strategy for treating patients with SCLC.

## MATERIALS AND METHODS

### Tumor specimens and survival data

125 consecutive patients with primary SCLC who had undergone complete surgical resection of the primary lung tumor from January 2003 through January 2013 at 16 institutions participating in either the Hokkaido Lung Cancer Clinical Study Group Trial (HOT) or the Fukushima Investigative Group for Healing Thoracic Malignancy (FIGHT) were analyzed [25]. Written informed consent was obtained only from patients who were still alive at the time of data accrual (from February 2013 through January 2014). All of the cases were included in the current study based on the following criteria: a complete surgical resection of primary tumors and a central re-review confirmed a pathological diagnosis of SCLC or combined SCLC according to the 2004 World Health Organization classification [26]. All individual data were obtained from medical records and de-identified. Stages were determined or reclassified according to the seventh version of the tumor-node-metastasis (TNM) staging system. The FFPE tissue block was cut into 20 to 30 sections, each 3 μm thick, and placed on glass slides for IHC.

### Immunohistochemical analysis

Notch1 and Numb expression was analyzed by immunohistochemistry. Archived sections were deparaffinized with xylene and rehydrated with graded concentrations of ethanol. For antigen retrieval, sections were placed in 10 mM citrate buffer, pH 6.0, and heated in a pressure cooker. Next, the sections were immersed in methanol containing 3% hydrogen peroxide for 10 minutes to block endogenous peroxidase activity, and were then incubated with normal goat serum to block nonspecific antibody binding sites. The sections were reacted consecutively with rabbit monoclonal anti-Notch1 antibody (1:400) (#3608, Cell Signaling Technology, Danvers, MA) or rabbit polyclonal anti-Numb antibody (1:500) (ab14140, Abcam, Cambridge, UK), at 4°C overnight. Immunostaining was performed using the biotin-streptavidin immunoperoxidase method with 3,3-diaminobenzidine as a chromogen. Hematoxylin solution was used for counterstaining. One positive sample was included as a positive external control with each staining batch. Positive tumor cells were counted under high magnification (×400) in five random and non-overlapping fields (100 tumor cells per field, total of 500 tumor cells per specimen). Positivity was defined as moderate-to-strong staining intensity, ‘moderate’ being of similar intensity to the positive control and ‘strong’ being of even higher intense. Only membrane staining was considered as negative and cytoplasm and/or nuclear staining were considered as positive staining in the evaluation of activated Notch1 expression. Immunohistochemical evaluations were performed independently by two investigators (H.K. and J.S.) who were blinded to the status of other immunohistological and clinical data, using a BX 40 microscope (Olympus, Tokyo, Japan). In the case of a discrepancy, the final results were decided by consensus.

### Statistical analysis

Associations between Notch1 or Numb expression and categorical variables were analyzed using chi-square tests or Fisher exact tests, as appropriate. Survival curves were estimated using the Kaplan-Meier method, and differences in survival distributions were evaluated using the log-rank test. Univariate and multivariate analyses using Cox proportional hazards modeling was applied to determine correlations between various factors and overall survival. The level of significance was set at P < 0.05. Statistical analyses were done using JMP software (JMP® Pro 11.0.0; SAS Institute Inc, USA).

## References

[1] Siegel R, Ma J, Zou Z, Jemal A Cancer Statistics, 2014. CA Cancer J Clin.

[2] Puglisi M, Dolly S, Faria A, Myerson JS, Popat S, O’Brien ME Treatment options for small cell lung cancer – do we have more choice?. Br J Cancer.

[3] Previs RA, Coleman RL, Harris AL, Sood AK Molecular pathways: translational and therapeutic implications of the Notch signaling pathway in cancer. Clin Cancer Res.

[4] Lobry C, Oh P, Aifantis I Oncogenic and tumor suppressor functions of Notch in cancer: it’s NOTCH what you think. J Exp Med.

[5] Konishi J, Kawaguchi KS, Vo H, Haruki N, Gonzalez A, Carbone DP, Dang TP Gamma-secretase inhibitor prevents Notch3 activation and reduces proliferation in human lung cancers. Cancer Res.

[6] Westhoff B, Colaluca IN, D'Ario G, Donzelli M, Tosoni D, Volorio S, Pelosi G, Spaggiari L, Mazzarol G, Viale G, Pece S, Di Fiore PP Alterations of the Notch pathway in lung cancer. Proc Natl Acad Sci U S A.

[7] Sriuranpong V, Borges MW, Ravi RK, Arnold DR, Nelkin BD, Baylin SB, Ball DW Notch signaling induces cell cycle arrest in small cell lung cancer cells. Cancer Res.

[8] Wael H, Yoshida R, Kudoh S, Hasegawa K, Niimori-Kita K, Ito T Notch1 signaling controls cell proliferation, apoptosis and differentiation in lung carcinoma. Lung Cancer.

[9] Hassan WA, Yoshida R, Kudoh S, Hasegawa K, Niimori-Kita K, Ito T Notch1 controls cell invasion and metastasis in small cell lung carcinoma cell lines. Lung Cancer.

[10] Nishimoto Y, Okano H New insight into cancer therapeutics: induction of differentiation by regulating the Musashi/Numb/Notch pathway. Cell Res.

[11] Wakamatsu Y, Maynard TM, Jones SU, Weston JA (1999). NUMB localizes in the basal cortex of mitotic avian neuroepithelial cells and modulates neuronal differentiation by binding to NOTCH-1. Neuron.

[12] McGill MA, McGlade CJ Mammalian numb proteins promote Notch1 receptor ubiquitination and degradation of the Notch1 intracellular domain. J Biol Chem.

[13] Donnem T, Andersen S, Al-Shibli K, Al-Saad S, Busund LT, Bremnes RM Prognostic impact of Notch ligands and receptors in nonsmall cell lung cancer: coexpression of Notch-1 and vascular endothelial growth factor-A predicts poor survival. Cancer.

[14] Reedijk M, Odorcic S, Chang L, Zhang H, Miller N, McCready DR, Lockwood G, Egan SE High-level coexpression of JAG1 and NOTCH1 is observed in human breast cancer and is associated with poor overall survival. Cancer Res.

[15] Yeh TS, Wu CW, Hsu KW, Liao WJ, Yang MC, Li AF, Wang AM, Kuo ML, Chi CW The activated Notch1 signal pathway is associated with gastric cancer progression through cyclooxygenase-2. Cancer Res.

[16] Zhang H, Wang X, Xu J, Sun Y Notch1 activation is a poor prognostic factor in patients with gastric cancer. Br J Cancer.

[17] Jin MM, Ye YZ, Qian ZD, Zhang YB Notch signaling molecules as prognostic biomarkers for non-small cell lung cancer. Oncol Lett.

[18] George J, Lim JS, Jang SJ, Cun Y, Ozretić L, Kong G, Leenders F, Lu X, Fernández-Cuesta L, Bosco G, Müller C, Dahmen I, Jahchan NS Comprehensive genomic profiles of small cell lung cancer. Nature.

[19] Pece S, Serresi M, Santolini E, Capra M, Hulleman E, Galimberti V, Zurrida S, Maisonneuve P, Viale G, Di Fiore PP Loss of negative regulation by Numb over Notch is relevant to human breast carcinogenesis. J Cell Biol.

[20] Hong J, Liu Z, Zhu H, Zhang X, Liang Y, Yao S, Wang F, Xie X, Zhang B, Tan T, Fu L, Nie J, Cheng C The tumor suppressive role of NUMB isoform 1 in esophageal squamous cell carcinoma. Oncotarget.

[21] Kang Y, Ding M, Tian G, Guo H, Wan Y, Yao Z, Li B, Lin D Overexpression of Numb suppresses tumor cell growth and enhances sensitivity to cisplatin in epithelioid malignant pleural mesothelioma. Oncol Rep.

[22] Verdi JM, Bashirullah A, Goldhawk DE, Kubu CJ, Jamali M, Meakin SO, Lipshitz HD Distinct human NUMB isoforms regulate differentiation vs. proliferation in the neuronal lineage. Proc Natl Acad Sci U S A.

[23] Bani-Yaghoub M, Kubu CJ, Cowling R, Rochira J, Nikopoulos GN, Bellum S, Verdi JM A switch in numb isoforms is a critical step in cortical development. Dev Dyn.

[24] Misquitta-Ali CM, Cheng E, O'Hanlon D, Liu N, McGlade CJ, Tsao MS, Blencowe BJ Global profiling and molecular characterization of alternative splicing events misregulated in lung cancer. Mol Cell Biol.

[25] Yokouchi H, Ishida T, Yamazaki S, Kikuchi H, Oizumi S, Uramoto H, Tanaka F, Harada M, Akie K, Sugaya F, Fujita Y, Fukuhara T, Takamura K Prognostic impact of clinical variables on surgically resected small-cell lung cancer: Results of a retrospective multicenter analysis (FIGHT002A and HOT1301A). Lung Cancer.

[26] (2004). Pathology and Genetics of Tumours of the Lung, Pleura, Thymus and Heart Third Edition.

